# High serum resistin levels are associated with peripheral artery disease in the hypertensive patients

**DOI:** 10.1186/s12872-017-0517-2

**Published:** 2017-03-15

**Authors:** Bang-Gee Hsu, Chung-Jen Lee, Chiu-Fen Yang, Yu-Chih Chen, Ji-Hung Wang

**Affiliations:** 1 Division of Nephrology, Buddhist Tzu Chi General Hospital, Hualien, Taiwan; 2 School of Medicine, Tzu Chi University, Hualien, Taiwan; 3 Department of Nursing, Tzu Chi University of Science and Technology, Hualien, Taiwan; 4 Division of Cardiology, Buddhist Tzu Chi General Hospital, No. 707, Section 3, Chung-Yang Road, Hualien, 97002 Taiwan

**Keywords:** Peripheral arterial disease, Ankle-brachial index, Hypertension, Resistin

## Abstract

**Background:**

Hypertension is a risk factor for peripheral arterial disease (PAD). Subjects with PAD are at increased risk of future cardiovascular (CV) events. Resistin is involved in the pathological processes of CV diseases. The aim of this study is to investigate whether resistin level is correlated with PAD in hypertensive patients.

**Methods:**

One hundred and twenty-four hypertensive patients were enrolled in this study. Ankle-brachial index (ABI) values were measured using the automated oscillometric method. An ABI value < 0.9 defined the low ABI group. Anthropometric analysis with waist circumference and body mass index, and fasting serum levels of blood urea nitrogen, creatinine, glucose, total cholesterol, triglycerides, high-density lipoprotein cholesterol, low-density lipoprotein cholesterol, total calcium, phosphorus, and high-sensitivity C-reactive protein (hs-CRP) were measured using standard enzymatic automated methods. Serum levels of human resistin were determined using a commercially available enzyme immunoassay.

**Results:**

Eighteen hypertensive patients (14.5%) were included in the low ABI group. Hypertensive patients in the low ABI group were older (*p* = 0.043) and had higher serum creatinine (*p* < 0.001), high-sensitivity C-reactive protein (hs-CRP; *p* = 0.013), and resistin (*p* < 0.001) levels but a lower estimated glomerular filtration rate (*p* = 0.002) than patients in the normal ABI group. After the adjustment for factors that were significantly associated with PAD on multivariate logistic regression analysis, serum resistin (odds ratio [OR], 1.176; 95% confidence interval [CI], 1.028–1.345; *p* = 0.018) was also an independent predictor of PAD in hypertensive patients.

**Conclusions:**

A high serum resistin level is an independent predictor of PAD in hypertensive patients.

## Background

Peripheral arterial disease (PAD) is a reflection of systemic atherosclerotic disease, which affected 202 million people worldwide in 2010 [1]. Although PAD can be asymptomatic and subclinical, symptomatic PAD is associated with reduced functional capacity and decreased quality of life [2]. PAD is also associated with an increased risk of incident coronary and cerebrovascular disease morbidity and mortality [2, 3]. The ankle-brachial index (ABI), the ratio of the systolic blood pressure (SBP) at the ankle to that in the arm, is non-invasive and inexpensive and currently used to assess PAD [3]. In a systematic review study, ABI had a sensitivity of 97% and specificity of 89% for detecting significant arterial disease on angiography [4].

Resistin is an adipocyte-derived signaling cysteine-rich molecule consisting of 114 amino acids [5]. Resistin is involved in the pathological processes of cardiovascular (CV) diseases including inflammation, endothelial dysfunction, thrombosis, angiogenesis, and smooth muscle cell dysfunction [6]. Serum resistin levels were higher in PAD patients than in healthy controls and an independent risk factor of PAD in a Chinese study [7]. PAD has been known to increase the risk of CV morbidity and mortality, resistin is involved in the pathological processes of CV diseases, and hypertension is one of the risk factors of PAD [2, 3]. The aim of this study is to examine the relationship between serum resistin levels and PAD in hypertensive patients.

## Methods

### Patients

From January to December 2012, 124 hypertensive patients enrolled at a medical center in Hualien, Taiwan. The blood pressure (BP) of each patient was measured in the morning by trained staff using standard mercury sphygmomanometers with appropriate cuff sizes after sitting for at least 10 min. Systolic BP and diastolic BP were taken three times at 5-min intervals and averaged for analysis. Patients were regarded as having hypertension if they had a systolic BP ≥ 140 mmHg, diastolic BP ≥ 90 mmHg, or received any anti-hypertensive medication in the past 2 weeks. Patients were diagnosed with diabetes mellitus if their fasting plasma glucose level was ≥126 mg/dL or they had used oral hypoglycemic medications or insulin [8]. The Protection of the Human Subjects Institutional Review Board of Tzu-Chi University and Hospital approved this study. All patients provided informed consent prior to participating in this study. The patients were excluded if they had an acute infection, acute myocardial infarction, or pulmonary edema, had a history of carotid artery stenosis or stroke; used protease-activated receptor-1 antagonists or warfarin at the time of blood sampling; or declined to provide informed consent.

### Anthropometric analysis

Waist circumference was measured using a tape measure at the point between the lowest ribs and the hip bones with the hands on the hips. Participant weight was measured in light clothing and without shoes to the nearest 0.5 kg, while height was measured to the nearest 0.5 cm. Body mass index (BMI) was calculated as the weight in kilograms divided by the height in meters squared [9–11].

### Biochemical investigations

Fasting blood samples (approximately 5 mL) were immediately centrifuged at 3000 × *g* for 10 min. Serum levels of blood urea nitrogen (BUN), creatinine (Cre), fasting glucose, total cholesterol (TCH), triglycerides (TG), high-density lipoprotein cholesterol (HDL-C), low-density lipoprotein cholesterol (LDL-C), total calcium, phosphorus, and high-sensitivity C-reactive protein (hs-CRP) were measured using an autoanalyzer (COBAS Integra 800; Roche Diagnostics, Basel, Switzerland) [9–11]. Serum levels of human resistin (SPI-BIO; Montigny le Bretonneux, France) [12] and intact parathyroid hormone (iPTH; Diagnostic Systems Laboratories, Webster, TX, USA) [9–11] were determined using a commercially available enzyme immunoassay or enzyme-linked immunosorbent assay, respectively. The estimated glomerular filtration rate (eGFR) was calculated using the Chronic Kidney Disease Epidemiology Collaboration equation.

### ABI measurements

Using an oscillometric method, ABI values were measured using an ABI-form device (VaSera VS-1000; Fukuda Denshi Co, Ltd, Tokyo, Japan) that automatically and simultaneously measures BP in both arms and ankles [13]. With the participants lying in the supine position, occlusion and monitoring cuffs were placed tightly around the four extremities, and an electrocardiogram was recorded and the heart sounds were measured for at least 10 min. ABI was calculated as the ratio of the ankle SBP divided by the arm SBP, and the lower value of the ankle SBP was used for the calculation. We repeatedly measured these parameters for both legs of each participant and expressed the mean values. PAD was diagnosed based on an ABI < 0.9 [14]. In this study, left or right ABI values < 0.9 were used to define the low ABI group.

### Statistical analysis

Data were tested for normal distribution using the Kolmogorov–Smirnov test. Normally distributed data are expressed as mean ± standard deviation and comparisons between patients were performed using the Student’s independent *t*-test (two-tailed). Non-normally distributed data were expressed as medians and interquartile ranges and comparisons between patients were performed using the Mann–Whitney *U* test (TG, fasting glucose, iPTH, hs-CRP, and resistin). Data expressed as the number of patients were analyzed by the *χ* ^2^ test. Because TG, fasting glucose, iPTH, hs-CRP, and resistin was not normally distributed and underwent base 10 logarithmic transformations to achieve normality. Clinical variables that correlated with serum resistin levels in hypertensive patients were evaluated using univariate linear regression analysis. Variables that were significantly associated with resistin levels in hypertensive patients were tested for independence on multivariate forward stepwise regression analysis. Variables that were significantly associated with PAD were tested for independence on multivariate logistic regression analysis (adapted factors: smoking, age, Cre, eGFR, hs-CRP, and resistin). The receiver operating curve (ROC) was used to calculate the area under the curve (AUC) to identify the cutoff value of resistin predicting PAD in hypertensive patients. Data were analyzed using SPSS for Windows (version 19.0; SPSS Inc., Chicago, IL, USA). *P* values < 0.05 were considered statistically significant.

## Results

The clinical and laboratory characteristics of the 124 hypertensive patients are shown in Table 1. Sixty-three patients (50.8%) had diabetes mellitus and 99 patients (79.8%) had dyslipidemia. Eighteen hypertensive patients (14.5%) were included in the low ABI group. Patients in the low ABI group were older (*p* = 0.043) and had higher serum Cre (*p* < 0.001), hs-CRP (*p* = 0.013), and resistin (*p* < 0.001) levels but had lower eGFR (*p* = 0.002) levels than those in the normal ABI group. Current smokers among low ABI group were significantly higher than the normal ABI group (*p* = 0.031). The use of drugs included angiotensin-converting enzyme inhibitors (ACEi; *n* = 44; 35.5%), angiotensin receptor blockers (ARB; *n* = 67; 54.0%), β-blockers (*n* = 72; 58.1%), calcium channel blockers (CCB; *n* = 56; 45.2%), statins (*n* = 69; 55.4%), fibrate (*n* = 30; 24.2%), aspirin (*n* = 72; 58.1%), and clopidogrel (*n* = 30; 24.2%). There was no statistically significant difference based on gender, co-existing diabetes or dyslipidemia, or use of ACEi, ARB, β-blockers, CCB, statins, fibrate, aspirin, or clopidogrel between the two groups. The resistin levels also did not differ statistically based on gender, co-existing diabetes or dyslipidemia, or ACEi, ARB, β-blocker, CCB, statin, fibrate, aspirin, or clopidogrel use (Table 2).

**Table 1:** Clinical variables of the 124 hypertensive patients in the normal or low ankle brachial index group

Characteristic	All patients (*n* = 124)	Normal ABI group (*n* = 106)	Low ABI group (*n* = 18)	*p* value
Age (years)	64.40 ± 9.81	63.67 ± 9.00	68.72 ± 13.15	0.043*
Height (cm)	161.33 ± 8.84	161.75 ± 8.62	158.83 ± 9.92	0.197
Body weight (kg)	69.96 ± 12.49	70.51 ± 12.78	66.78 ± 10.39	0.243
Waist circumference (cm)	91.87 ± 9.00	91.63 ± 8.52	93.28 ± 11.65	0.476
Body mass index (kg/m^2^)	26.80 ± 3.72	26.84 ± 3.69	26.53 ± 3.96	0.740
Left ankle-brachial index	1.06 ± 0.11	1.09 ± 0.08	0.87 ± 0.10	<0.001*
Right ankle-brachial index	1.05 ± 0.12	1.08 ± 0.08	0.84 ± 0.10	<0.001*
Systolic blood pressure (mmHg)	134.63 ± 16.97	133.62 ± 16.11	140.56 ± 20.92	0.109
Diastolic blood pressure (mmHg)	74.10 ± 10.18	74.30 ± 10.46	72.94 ± 8.47	0.603
Total cholesterol (mg/dL)	174.18 ± 38.59	174.38 ± 40.54	173.00 ± 24.90	0.889
Triglyceride (mg/dL)	128.00 (93.25–177.25)	122.00 (90.75–178.25)	137.00 (114.00–172.50)	0.383
HDL-C (mg/dL)	45.19 ± 12.81	45.38 ± 12.85	44.11 ± 12.88	0.700
LDL-C (mg/dL)	103.19 ± 30.46	103.03 ± 32.11	104.11 ± 18.47	0.890
Fasting glucose (mg/dL)	111.00 (96.25–137.75)	111.00 (96.75–137.25)	110.00 (94.50–154.00)	0.980
Blood urea nitrogen (mg/dL)	17.36 ± 6.11	16.99 ± 5.75	19.56 ± 7.76	0.100
Creatinine (mg/dL)	1.13 ± 0.33	1.09 ± 0.28	1.38 ± 0.47	<0.001*
eGFR (mL/min)	67.81 ± 20.37	70.10 ± 18.81	54.33 ± 24.35	0.002*
Total calcium (mg/dL)	9.16 ± 0.37	9.15 ± 0.38	9.20 ± 0.33	0.560
Phosphorus (mg/dL)	3.55 ± 0.51	3.54 ± 0.53	3.57 ± 0.38	0.854
Intact parathyroid hormone (pg/mL)	46.65 (33.53–61.95)	46.30 (32.40–59.85)	51.20 (36.45–77.78)	0.272
hs-CRP (mg/dL)	0.21 (0.15–0.29)	0.20 (0.14–0.27)	0.28 (0.20–0.55)	0.013*
Resistin (ng/mL)	7.19 (5.21–10.33)	6.67 (4.77–8.59)	11.79 (9.94–16.72)	<0.001*
Male, n (%)	82 (66.1)	71 (67.0)	11 (61.1)	0.627
Diabetes, n (%)	63 (50.8)	52 (49.1)	11 (61.1)	0.344
Dyslipidemia, n (%)	99 (79.8)	85 (80.2)	14 (77.8)	0.814
Smoking, n (%)	11 (8.9)	7 (6.6)	4 (22.2)	0.031*
ACE inhibitor use, n (%)	44 (35.5)	39 (36.8)	5 (27.8)	0.460
ARB use, n (%)	67 (54.0)	57 (53.8)	10 (55.6)	0.888
β-blocker use, n (%)	72 (58.1)	60 (56.6)	12 (66.7)	0.424
CCB use, n (%)	56 (45.2)	49 (46.2)	7 (38.9)	0.563
Statin use, n (%)	69 (55.4)	58 (54.7)	11 (61.1)	0.614
Fibrate use, n (%)	30 (24.2)	28 (26.4)	2 (11.1)	0.161
Aspirin, n (%)	72 (58.1)	64 (60.4)	8 (44.4)	0.205
Clopidogrel, n (%)	30 (24.2)	27 (25.5)	3 (16.7)	0.420

Values for continuous variables are shown as mean ± standard deviation after analysis by Student’s *t*-test; variables not normally distributed are shown as median and interquartile range after analysis by the Mann–Whitney *U* test; values are presented as number (%) and analysis after analysis by the chi-square test

*ABI* ankle brachial index, *HDL-C* high-density lipoprotein cholesterol, *LDL-C* low-density lipoprotein cholesterol, *eGFR* estimated glomerular filtration rate, *hs-CRP* high-sensitivity C-reactive protein, *ACE* angiotensin-converting enzyme, *ARB* angiotensin-receptor blocker, *CCB* calcium-channel blocker

*Values of *p* < 0.05 were considered statistically significant

**Table 2:** Clinical characteristics and serum resistin levels of 124 hypertensive patients

Characteristic		Number (%)	Resistin (ng/mL)	*p* value
Sex	Male	82 (66.1)	7.26 (5.10–10.11)	0.631
	Female	42 (33.9)	6.95 (5.60–11.98)	
Diabetes	No	61 (49.2)	6.86 (5.12–10.01)	0.472
	Yes	63 (50.8)	7.41 (5.24–10.89)	
Dyslipidemia	No	25 (20.2)	7.23 (5.14–9.55)	0.818
	Yes	99 (79.8)	7.18 (5.20–10.89)	
ACE inhibitor	No	80 (64.5)	7.17 (5.14–10.81)	0.938
	Yes	44 (35.5)	7.22 (5.26–10.16)	
ARB	No	57 (46.0)	7.20 (4.91–10.44)	0.833
	Yes	67 (54.0)	7.18 (5.24–10.19)	
β-blocker	No	52 (41.9)	6.95 (5.34–9.22)	0.491
	Yes	72 (58.1)	7.39 (4.94–11.74)	
CCB	No	68 (54.8)	7.04 (4.67–10.16)	0.498
	Yes	56 (45.2)	7.42 (5.40–10.79)	
Statin	No	55 (44.6)	7.41 (5.68–10.55)	0.260
	Yes	69 (55.4)	6.88 (4.77–10.02)	
Fibrate	No	94 (75.8)	7.33 (5.36–10.51)	0.253
	Yes	30 (24.2)	6.76 (4.56–8.56)	
Aspirin	No	52 (41.9)	8.90 (5.78–10.24)	0.525
	Yes	72 (58.1)	7.88 (4.92–10.16)	
Clopidogrel	No	94 (75.8)	8.53 (5.36–10.17)	0.253
	Yes	30 (24.2)	7.61 (4.56–8.56)	

Data are expressed as median and interquartile range after analysis by the Mann–Whitney *U* test

*ACE* angiotensin-converting enzyme, *ARB* angiotensin-receptor blocker, *CCB* calcium-channel blocker

*Values of *p* < 0.05 were considered statistically significant after analysis by the Mann–Whitney *U* test

Uni- and multivariate linear analyses of the clinical variables associated with serum resistin levels in hypertensive patients are shown in Table 3. Waist circumference (*r* = 0.261; *p* = 0.003), TCH (*r* = 0.182; *p* = 0.043), LDL-C (*r* = 0.190; *p* = 0.035), BUN (*r* = 0.237; *p* = 0.008), Cre (*r* = 0.298; *p* = 0.001), and logarithmically transformed hs-CRP (log-hs-CRP, *r* = 0.294; *p* = 0.001) were positively correlated, while eGFR (*r* = −0.278; *p* = 0.002) was negatively correlated with serum resistin levels in hypertensive patients. Multivariate forward stepwise linear regression analysis of the variables significantly associated with fasting serum resistin levels revealed that waist circumference (β = 0.204; *p* = 0.014), LDL-C (β = 0.165; *p* = 0.044), Cre (β = 0.243; *p* = 0.004), and log-hs-CRP (β = 0.236; *p* = 0.005) were independent predictors of resistin values for hypertensive patients.

**Table 3:** Correlation between serum resistin levels and clinical variables among the 124 hypertensive patients

Variable	Log-Resistin (ng/mL)			
	Univariate		Multivariate	
	r	*p*	Beta	*p*
Age (years)	0.135	0.135	-	-
Height (cm)	0.006	0.949	-	-
Body weight (kg)	−0.117	0.197	-	-
Waist circumference (cm)	0.261	0.003*	0.204	0.014*
Body mass index (kg/m^2^)	−0.149	0.098	-	-
SBP (mmHg)	−0.102	0.261	-	-
DBP (mmHg)	0.044	0.629	-	-
Total cholesterol (mg/dL)	0.182	0.043*	-	-
Log-Triglyceride (mg/dL)	0.015	0.872	-	-
HDL-C (mg/dL)	0.061	0.501	-	-
LDL-C (mg/dL)	0.190	0.035*	0.165	0.044*
Log-glucose (mg/dL)	−0.059	0.517	-	-
Blood urea nitrogen (mg/dL)	0.237	0.008*	-	-
Creatinine (mg/dL)	0.298	0.001*	0.243	0.004*
eGFR (mL/min)	−0.278	0.002*	-	-
Total calcium (mg/dL)	−0.094	0.302	-	-
Phosphorus (mg/dL)	−0.109	0.228	-	-
Log-iPTH (pg/mL)	0.146	0.106	-	-
Log-hs-CRP (mg/dL)	0.294	0.001*	0.236	0.005*

Data of resistin, triglyceride, glucose, iPTH, and hs-CRP levels showed a skewed distribution and were, therefore, log-transformed before analysis

Analysis of the data was done using the univariate linear regression analyses or multivariate stepwise linear regression analysis (adopted factors: waist circumference, total cholesterol, blood urea nitrogen, creatinine, eGFR, and hs-CRP)

*SBP* systolic blood pressure, *DBP* diastolic blood pressure, *HDL-C* high density lipoprotein-cholesterol *LDL-C* low-density lipoprotein cholesterol, *eGFR* estimated glomerular filtration rate, *iPTH* intact parathyroid hormone, *hs-CRP* high-sensitivity C-reactive protein

*Values of *p* < 0.05 were considered statistically significant

**Fig. 1:**
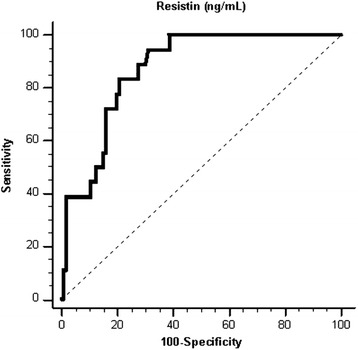
The area under the receiver operating characteristic curve indicates the diagnostic power of resistin for predicting peripheral artery disease of hypertensive patients

Adjustment of the factors significantly associated with PAD (smoking, age, Cre, eGFR, hs-CRP, and resistin) on multivariate logistic regression analysis revealed that increased serum resistin level (odds ratio [OR], 1.176; 95% confidence interval [CI], 1.028–1.345; *p* = 0.018) was an independent predictor of PAD in hypertensive patients (Table 4); plotting of the ROC curve for PAD prediction revealed that the AUC for resistin was 0.870 (95% CI, 0.798–0.923; *p* < 0.001) (Fig. 1).

**Table 4:** Multivariate logistic regression analysis of the factors correlated to peripheral artery disease among the 124 hypertensive patients

Variable	Odds ratio	95% Confidence interval	*p* value
Resistin (ng/mL) (each increase of 1 ng/mL)	1.176	1.028-1.345	0.018*

Analysis of the data was done using multivariate logistic regression analysis (adopted factors: smoking, age, creatinine, estimated glomerular filtration rate, high-sensitivity C-reactive protein, and resistin)

*Values of *p* < 0.05 were considered statistically significant

## Discussion

The results of this study showed that hypertensive patients with PAD were older and had higher serum Cre, hs-CRP, and resistin levels but a lower eGFR. Serum resistin level was an independent clinical predictor of PAD in hypertensive patients after multivariate analysis. Among these patients, waist circumference and LDL-C, Cre, and log-hs-CRP levels were the independent predictors of resistin values.

PAD is a result of the blockage of the arteries supplying blood to the brain, visceral organs, and the limbs and usually occurs secondarily to atherosclerosis [15, 16]. The prevalence of PAD increases sharply with age, and it affects a substantial proportion of the elderly population [1–3]. Moreover, men and women from low- and middle-income countries have modestly lower PAD rates than those in high-income countries [1]. The prevalence in high-income countries at age 45–49 years was 5.28% in women and 5.41% in men; at age 85–89 years, it was 18.38% in women and 18.83% in men. The prevalence in men was lower in low- and middle-income countries than in high-income countries (2.89% at 45–49 years and 14.94% at 85–89 years) [1]. The prevalence in Asian-Americans at age 60–69 years was 2.6% in women and 10.2% in men; at age ≥ 80 years, it was 17.1% in women and 13.8% in men [17].

Most studies found a significant independent association between hypertension and PAD. The odds ratio of hypertension for PAD was 1.32–2.20 [3]. The mean age in this study was 64.4 years and the prevalence was 14.5% in hypertensive patients; of note, hypertensive patients with PAD tended to be older. Male gender, smoking, and comorbid diabetes or dyslipidemia are established risk factors for PAD [2, 3, 16]. Our results also noted current smokers among low ABI group were significantly higher than the normal ABI group. Our study did not find statistically significant differences in gender or co-existing diabetes or dyslipidemia in hypertensive patients, possibly due to an insufficient sample size.

A high prevalence of PAD is noted in the US population > 40 years old with renal insufficiency, and even after the adjustment for important confounding factors, persons with renal insufficiency are still more than twice as likely to have an ABI < 0.9 according to the National Health and Nutrition Examination Survey 1999–2000 [18]. Renal insufficiency was also independently associated with future PAD events among postmenopausal women with coronary heart disease. The hazard ratio for PAD in women with a Cre clearance 30–59 mL/min/1.73 m^2^, < 30 mL/min/1.73 m^2^ is 1.63, 3.24 compared with persons with a Cre clearance ≥ 60 mL/min/1.73 m^2^ [19]. Our results also noted that hypertensive patients in the low ABI group had higher serum Cre levels and a lower eGFR. Inflammatory markers such as CRP are associated with PAD in many studies [2, 3]. Wildman et al. noted that the adjusted OR of PAD associated with the highest versus the lowest quartile of CRP was 2.14 in a sample of 4,787 participants aged > 40 years in the National Health and Nutrition Examination Survey 1999–2002 [20]. CRP was associated with fatal and nonfatal cardiovascular disease events as well as nonfatal PAD events in 18,450 apparently healthy participants in the European Prospective Investigation into Cancer and Nutrition-Norfolk cohort [21]. The hs-CRP level was also statistically significantly more correlated in PAD patients, who were at higher risk for CV morbidity and mortality in the presence of an elevated hs-CRP level [22]. Similar to these studies, our study revealed that hypertensive patients with a low ABI had higher serum hs-CRP levels.

Resistin is an adipokine involved in glucose homeostasis, lipid metabolism, and insulin action [23–25]. Circulating resistin levels have been positively associated with central obesity as well as insulin resistance in rodents; however, their significance remained controversial in human studies [25]. Resistin was positively associated with waist circumference and insulin resistance and inversely associated with TCH, HDL-C, and LDL-C after adjustment for age, gender, and BMI in a study with 1,508 Finnish subjects aged 45–74 years [26]. In an Indian study, plasma resistin levels were also positively associated with waist circumference and insulin resistance. Moreover, plasma resistin levels were strongly positively correlated with TCH, HDL-C, and LDL-C levels [27]. Our results also noted that serum resistin levels were positively associated with waist circumference and LDL-C level after multivariate linear regression analysis. Human resistin was shown to be predominantly expressed in peripheral blood mononuclear cells, macrophages, and bone marrow cells by analyses of resistin gene expression across a wide array of human tissues [5, 23].

Human resistin is an inflammatory biomarker that functions through the nuclear factor-κB and other signaling pathways that induce pro-inflammatory processes [6, 25]. Plasma resistin levels are positively associated with CRP and predictive of coronary atherosclerosis in humans [28]. A high serum resistin level is associated with a low eGFR in patients with type 2 diabetes and those with chronic kidney disease [29, 30]. High plasma resistin levels were independently associated with a greater risk of kidney function decline in middle-aged and elderly Chinese patients [31]. Our results showed that serum Cre and log-hs-CRP levels were positively correlated with serum resistin levels in hypertensive patients after multivariate analysis.

Resistin has a pathogenic role in the development and progression of atherosclerosis [6, 23]. Atherosclerosis is the major cause of PAD by arterial stenosis or occlusion, particularly of the lower extremities [15, 16]. Our study revealed that hypertensive patients in the low ABI group had higher serum resistin levels compared to those in the normal ABI group. Adjustment of the confounding factors on the multivariate logistic regression analysis revealed that an increased serum resistin level was an independent predictor of PAD in hypertensive patients.

Use of the ABI test to diagnose PAD has several limitations. Occlusive disease distal to the ankle is not detected by the ABI test, which is also sensitive to the patient’s height [3]. The other limitation of this study is that it was an observational, single-center study with a small sample of hypertensive participants. The use of anti-hypertensive drugs such as ACEi or ARB or of statins is beneficial for reducing major adverse cardiovascular events in patients with PAD and hypertension [32]. However, evidence of the use of various anti-hypertensive drugs in people with PAD is poor [33]. Another limitation is that pharmacological interventions have been shown to influence resistin levels in humans. The use of amlodipine, bisoprolol, or indapamide resulted in significantly lower plasma resistin concentrations after 6 weeks of treatment [34]. Statin therapy did not result in any significant changes in plasma resistin concentrations in a meta-analysis [35]. The current results did not show a correlation between anti-hypertensive drugs, statins, fibrates, aspirin, or clopidogrel and serum resistin levels. Further studies are required to elucidate the relationship between medication use and resistin levels in hypertensive patients.

## Conclusions

This study showed that a higher resistin level was an independent predictor of the development of PAD in hypertensive patients. In addition, waist circumference, LDL-C, Cre, and log-hs-CRP were positively associated with resistin values in these patients.

## Abbreviations

ABI: Ankle-brachial index
ACEi: Angiotensin-converting enzyme inhibitors
ARB: Angiotensin receptor blockers
AUC: Area under the curve
BMI: Body mass index
BP: Blood pressure
BUN: Blood urea nitrogen
CCB: Calcium channel blockers
CI: Confidence interval
Cre: Creatinine
CV: Cardiovascular
eGFR: Estimated glomerular filtration rate
HDL-C: High-density lipoprotein cholesterol
hs-CRP: High-sensitivity C-reactive protein
iPTH: Intact parathyroid hormone
LDL-C: Low-density lipoprotein cholesterol
OR: Odds ratio
PAD: Peripheral arterial disease
ROC: Receiver operating curve
TCH: Total cholesterol
TG: Triglyceride
